# A Comprehensive Review on Ensemble Solar Power Forecasting Algorithms

**DOI:** 10.1007/s42835-023-01378-2

**Published:** 2023-01-12

**Authors:** Negar Rahimi, Sejun Park, Wonseok Choi, Byoungryul Oh, Sookyung Kim, Young-ho Cho, Sunghyun Ahn, Chulho Chong, Daewon Kim, Cheong Jin, Duehee Lee

**Affiliations:** 1 Deptartment of Electrical and Electronic Engineering, Konkuk University, Seoul, South Korea; 2 EINS S&C, Seoul, South Korea

**Keywords:** Ensemble methods, Solar forecasting, Cooperative ensemble forecasting

## Abstract

With increasing demand for energy, the penetration of alternative sources such as renewable energy in power grids has increased. Solar energy is one of the most common and well-known sources of energy in existing networks. But because of its non-stationary and non-linear characteristics, it needs to predict solar irradiance to provide more reliable Photovoltaic (PV) plants and manage the power of supply and demand. Although there are various methods to predict the solar irradiance. This paper gives the overview of recent studies with focus on solar irradiance forecasting with ensemble methods which are divided into two main categories: competitive and cooperative ensemble forecasting. In addition, parameter diversity and data diversity are considered as competitive ensemble forecasting and also preprocessing and post-processing are as cooperative ensemble forecasting. All these ensemble forecasting methods are investigated in this study. In the end, the conclusion has been drawn and the recommendations for future studies have been discussed.

## Introduction

**Fig. 1:**
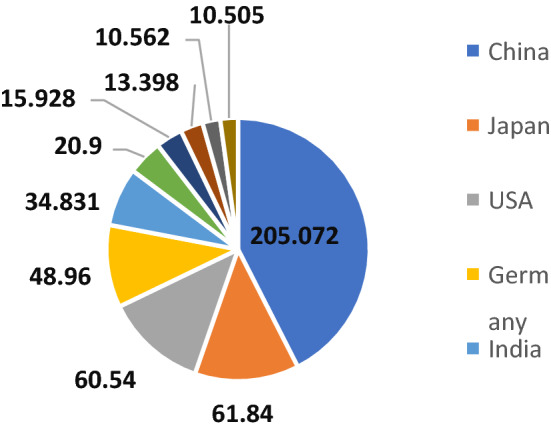
Top 10 countries in 2019 based on total PV installed capacity (GW) [3]

Over the past decades, the installation and use of renewable energy resources have been increased due to environmental issues such as greenhouse gas emission and the reduction of fossil fuels [1]. Solar energy has been considered as one of the promising renewable energy resources that is able to supply global energy demand [2]. According to the International Renewable Energy Agency (IRENA), the top ten countries with the highest installed solar PV capacity are shown in Fig. 1 [3].

According to Renewables 2015-Global Status Report, the global capacity of PV was increased from 3.7 GW to 7 GW between 2004 and 2007 and promoted to 40 GW by 2010. In 2015, the global installed capacity of solar PV was around 177 GW [4]. From 2016 to 2018, based on IRENA, there was a significant rose in this amount, and it was reached about 580 GW in 2019 [5]. It has been reported that in the first half of 2020, when the COVID-19 pandemic began, the renewable energy sector experienced a turbulent but in the second half of 2020, the PV sector set a new record for new capacity which bring the solar sector to its highest level of more than 800 Gigawatts [6].

However, the stochastic and unpredictable nature of solar energy led to a number of challenges such as voltage fluctuations and uncertainties in a power grid that make it difficult to maintain a balance between power generation and load demand [7]. For example, in cloudy days, the amount of solar irradiation received by PV modules has many fluctuations because of the cloud’s movement, and the random fluctuations have a significant effect on the solar PV output. Various solutions including power system, scheduling battery reserves, demand response, backup generators, and dispatches have been proposed to prevent the above problems. However, several limitations exist in all these solutions. There is a restriction for the power decline rate of backup generators which caused by the unit ramp rate resulted in difficulties to meet the incremental power generation need. However, due to the storage capacity restrictions and the costs of battery reserves, massive-scale energy storage is still difficult to realize. In addition, there is a difficulty to achieve demand response technologies based on the lack information on behavior of electricity consumption by consumers. Besides, the efficiency of the above-mentioned solutions largely depends on forecasting accuracy (with different time horizons) [8].

There are two important aspects of accurate forecasting: reducing the negative effect of random PV power on the power grid and providing and predicting PV power output data for grid operators. Hence, there is a need to forecast the output power of solar systems for the efficient operation of the power grid. The optimal management of a power system and scheduling is important for estimation of the reserves. It should be noted that the solar forecasting becomes important due to the substantial increase of solar power generation worldwide [9].

Recently, the ensemble forecasting was recommended for solar power forecasting. In the ensemble forecasting, many different predictions from different forecasting are averaged. Averaging predictions can reduce server biases when weather data in outliers, so it can avoid the worst predictions. However, it takes a lot of computational time to run the ensemble forecasting model since several forecasting models should be simulated at the same time. However, this computational time can be further reduced when the parallel computing environment is used. In this study, we organize several ensemble solar power forecasting algorithms.

For forecasting methods of PV systems, several review papers have been published during the last 5 years with different scopes. Their focus was ensemble methods, PV output power forecasting different PV forecasting methods, probabilistic forecasting in solar PV [10], hybrid models for solar radiation forecasting, post-processing in solar forecasting, different methods for forecasting solar irradiance. Hence, there is no updated review with a focus on ensemble methods only. Therefore, we reviewed the ensemble methods for solar irradiation forecasting which are divided into main categories competitive (data diversity and parameter diversity) and Cooperative forecasting (pre-processing and post-processing). The recent research papers that have been selected which have been published in the last four years (2018–2022) were reviewed. The above-mentioned papers focused on reviewing solar forecasting methods. In this paper, the focus was ensemble forecasting methods and their classifications in recent years.

For the ensemble forecasting, there are two topics, namely, solar power forecasting and solar irradiance forecasting which are known as solar forecasting. Meanwhile, they have strongly correlated each other and cannot be separated [11]. It should be noted that there is a strong impact of solar irradiance on the accuracy of solar power forecasts for the power production systems of various sorts. If a forecaster wants to achieve high-quality solar power forecasts, the ability to produce and use irradiance forecasts is essential. In other words, the best solar power prediction is always obtained by irradiance forecasts generation and conversion of those irradiance forecasts into power forecasts through a model chain. Therefore, there is not an intrinsic value for irradiance forecast [12].

## Parameters Affecting Solar Power Forecasting

Forecast horizon, weather classification, error metrics, data processing affect the output of solar forecasting.

### Forecast Horizon

The forecast horizon can be considered as the period of time in the future (time duration between actual and effective time) in which the forecasting should be done [13]. Forecasting horizon can be classified into four categories including (1) very short-term, (2) short-term, (3) medium-term, and (4) long-term [14]. These categories have summarized in Table 1 based on their forecasting period and related applications in solar energy systems. However, there is still no universal classification criterion [15].

**Table 1:** Different types of forecasting horizon and its applications [15]

Forecasting horizon	Forecasting period	Applications
Very short-term	From seconds to 1 min ahead	– Electricity market – Monitoring of real-time electricity dispatch – PV storage control
Short-term	Several hours ahead	Unit commitment
Medium-term	One week or within a month ahead	Scheduling of power systems maintenance (solar energy integrated power or conventional systems)
Long-term	Months or Years ahead	– Electricity pricing – Load forecasting

Furthermore, the researchers usually prefer to use another categories to describe forecast horizon including intra-hour, intra-day, and day-ahead which have overlapping with short, medium, and long-term forecast horizons [16].

#### Intra-Hour

Intra-hour overlaps with very short-term and short-term horizon categories and it also shows the forecast horizon from a few seconds to an hour. It is used for operating regulation reserves, storage system optimization and ensuring grid quality and stability. Such prediction methods can be applied in high solar penetration areas such as island grids with spans of 1 to 6 h.

#### Intra-Day

This forecast horizon is used for 1–6 h and also overlaps with short and medium categories and its application is in electricity trading outside the standard grid and control of electric loads [13].

#### Day-Ahead

Day-ahead Forecasts spanning 6–48 h overlaps with long-term and medium horizons. Similar models have been used in unit commitment and utilities planning [13]. In general, before designing PV power forecasting model, the appropriate forecast time horizon should be selected because the accuracy of a predicted model depends on the forecast time horizon [17]. It has been proved that by increasing the forecasting horizon, the accuracy of forecasting model (both single and ensemble models) will reduce. This is because the correlation between cloud cover and solar irradiance, which cannot be accurately predicted for long periods of time. In addition, for power system planning, long time horizon forecast is suitable while for PV output forecasting, intra-hour and intra-day forecast horizons work better [13]. In other words, the model performance decreases with long-time ahead forecasting (regardless of model type) while the performance would be increased with short time ahead forecasting [18].

### Weather Classification

It is obvious that the PV output is directly related to solar irradiance and the accuracy solar irradiance forecasting models is strongly affected by meteorological factors such as cloud cover, temperature, humidity and wind speed. So, climate change and different weather types have significant effect on PV system output power. Therefore, to enhance the prediction performance, weather condition is an effective step especially for solar irradiance forecasting [19]. Typically, there are two types of weather condition including the normal (ideal) weather type (sunny days), and abnormal (non-ideal) weather types (rainy, foggy and windy days) [20]. According to some studies [19–21], the PV output power increases in the ideal weather condition (on sunny days) but it decreases in non-ideal conditions.

### Error Metrics

In different steps of model development, evaluation error metrics are one of the important parameters. In these kinds of metrics, the comparison of the actual solar irradiance and predicted solar irradiance are considered [22]. It should be noticed that there are different units for performance of metrics while W/m2 has been used as unit for the statistical error of solar radiation [18]. The most used evaluation metrics for statistical measures are listed as below:

#### Mean Bias Error (MBE)

Mean bias error (MBE): It shows the average bias of a forecasting model:

1 $$MBE = \frac{1}{N}\sum\limits_{i = 1}^{N} {\left( {y_{i(pred)} - y_{i(act)} } \right)}$$

The larger value of MBE shows the larger forecast bias. A positive value of MBE indicates over-forecasting, while a negative value means under-forecasting [23].

#### Mean Absolute Error (MAE)

Mean absolute error (MAE): This error is defined as the average of the absolute difference between forecasted and actual solar irradiance values. This metric is suitable for uniform forecast errors as equal weight will be given to all discrepancies in the data, and it is also used for both regression problems and evaluation of overall forecast accuracy. Generally, the smaller MAE is better in forecasting.

2 $$MAE = \frac{1}{N}\sum\limits_{i = 1}^{N} {\left| {y_{i(pred)} - y_{i(act)} } \right|}$$

where $y_{i(act)}$ is the actual solar irradiance, N is the total number of observations and $y_{i(pred)}$ is the predicted solar irradiance [22, 24].

#### Mean Square Error (MSE)

Mean square error (MSE): It is calculated by averaging the square of difference between the actual and predicted solar irradiance values [25].

3 $$MSE = \frac{1}{N}\sum\limits_{i = 1}^{N} {\left( {y_{i(pred)} - y_{i(act)} } \right)^{2} }$$

#### Root Mean Square Error (RMSE)

Root mean square error (RMSE) is for calculating this metric, the square root for the average of the squared differences of predicted and actual solar irradiance values is considered. RMSE is known as the most appreciated performance evaluation metrics which the outliers in the data can be eliminated and identified by using this error [25]. Also, as RMSE emphasizes the larger errors, it can be used as the main error metric [26].

4 $$RMSE = \sqrt {\frac{1}{N}\sum\limits_{i = 1}^{N} {\left( {y_{i(pred)} - y_{i(act)} } \right)^{2} } }$$

#### Normalized RMSE (nRMSE)

Normalized RMSE (nRMSE) is the overall deviations of larger datasets nRMSE can be calculated.

5 $$nRMSE = \frac{{\sqrt {\frac{1}{N}\sum\nolimits_{i = 1}^{N} {\left( {y_{i(pred)} - y_{i(act)} } \right)^{2} } } }}{{\overline{y}}}$$

where ($\overline{y}$) denotes the mean of the actual solar irradiance [27].

#### Mean Absolute Percentage Error (MAPE)

Mean absolute percentage error (MAPE) is appropriate to evaluate uniform prediction errors like MAE however it can be calculated by the difference between each predicted and actual observation divide by the actual observed value.

6 $$MAPE = \frac{1}{N}\sum\limits_{i = 1}^{N} {\frac{{\left| {\mathop y\nolimits_{i(pred)} - \mathop y\nolimits_{i(act)} } \right|}}{{\mathop y\nolimits_{i(act)} }}}$$

#### Determination Coefficient (${{\varvec{R}}}^{2}$)

Determination coefficient (R^2) is used to extract the information of the determination between the forecasted and the real values and the ranges is between 0 and 1 [28].

#### Skill Score ($\mathbf{S}\mathbf{S}$)

Skill score (SS) measures the performance comparison of a forecast model to a benchmark model is defined as S score and calculated as follows:

7 $$S = 1 - \frac{{\mathop {RMSE}\nolimits_{{forecast\;{\text{model}}}} }}{{\mathop {RMSE}\nolimits_{{benchmark\;{\text{model}}}} }}$$

A good forecast model has a SS score of 1. In addition, SS zero shows a model with forecast error equal to the benchmark model while a negative SS score is a forecast with higher forecast error [1].

### Model Inputs

Inputs are considered as a key factor in forecasting and have a significant effect on prediction accuracy. Generally, inappropriate inputs can cause forecast errors in a system, for example, time delay, cost, and computational complexity [13]. In addition, the correlation of the input and output values affects the performance of a forecasting model. Therefore, the correlation of PV power output with the different meteorological inputs, such as wind speed and direction, module temperature, atmospheric temperature, solar irradiance, and humidity is important [17]. Among these parameters, solar irradiance is the most significant input for the forecast and the accuracy of solar irradiance measurement affects the precision of solar power generation [29]. Demonstrated the highest influence in solar power generation related to the intensity of solar irradiance. In [30] a SVR-based forecasting model was proposed for PV power generation forecasting. In this study, the data of three different PV plants, in Malaysia, including the actual PV power generation data and meteorological data (wind speed atmospheric temperature and solar irradiance) were used and PV power output was received only from 8:00 AM until 19:00 PM. Also, PV output for a specific day and the pattern of solar irradiance was presented and the correlation coefficient (R^2 = 0.9888) was obtained. The correlation coefficient indicated that there is a strong correlation between solar irradiance and PV power output. It means that if PV power increases, then the irradiance will be increased, and vice versa. Other studies also showed that the PV power generation are correlated with other meteorological variables, such as clouds, module temperature, ambient temperature, and wind speed. Also, fluctuations in sunlight intensity caused by several parameters such as dissipation, cloud motion, deformation and birth which affect PV output [31]. In another study by [32], PV output variation with solar irradiance was analyzed during clear day, partially cloudy and cloudy day. Based on the results, in comparison with to partially cloudy and clear day, there was a high fluctuations degree with solar irradiance were observed in cloudy days.

Another input variables linked together are wind speed, ambient and module temperature. The temperature of the module and its level of efficiency depend on the ambient temperature and the amount of absorbed radiation, as well as the wind speed. When the ambient temperature increases (It is related to cloud cover), the module temperature rises and its efficiency also increases, while the wind speed reduces the module temperature and heat loss [33]. It is concluded that there was a weak correlation between atmospheric temperature and PV power output while there was an extremely weak correlation between wind speed and PV power output. In contrast, there was a strong correlation between module temperature and PV power output [30].

## Classification of Solar Forecasting Algorithms

This section classifies solar forecasting algorithms. The solar forecasting algorithms are categorized into three main models, such as ensemble methods physical and statistical time-series.

Group one is statistical models, which are based on historical data as input for prediction and the internal behavior of the model does not have any effect on it [34]. In other words, to reconstruct the hourly irradiance and metrological parameters statistical models can be used [35]. Different techniques are applied for statistical time series such as support vector machine and Markov chain, regression model, and artificial neural network [34].

The second group generated solar power output from external data such as temperature and solar irradiance and their physical relationship. The generation of irradiance forecast between many meteorological parameters would be achieved by using mathematical models. It can be said that the results are highly dependent on the accurate input data of the model [36]. This group can be categorized into two subsections of Empirical models and Numerical weather prediction (NWP) [37].

**Fig. 2:**
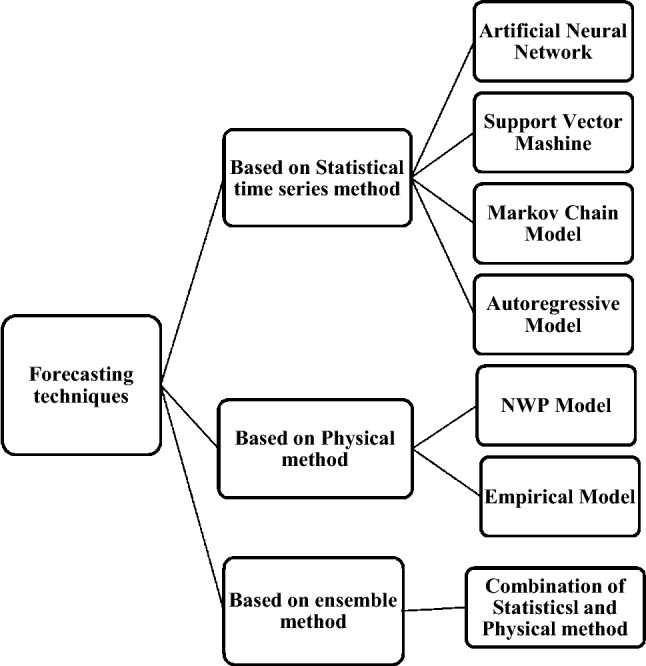
Forecasting techniques based on three major models [39]

The last group is hybrid or ensemble methods which used the combination of physical and statistical methods with unique features to solve the limitations of an individual model. By these models, at the same time, the forecasting performance increases, and the model would be improved. Figure 2 shows these forecasting methods [38].

For forecasting methods of PV systems, several review papers have been published during the last 5 years with different scopes. Their focus was ensemble methods, PV output power forecasting [14, 32] different PV forecasting methods, probabilistic forecasting in solar PV [40], hybrid models for solar radiation forecasting [41], post-processing in solar forecasting [42], different methods for forecasting solar irradiance [24]. Hence, there is no updated review with a focus on ensemble methods only. Therefore, we reviewed the ensemble methods for solar irradiation forecasting which are divided into main categories competitive (data diversity and parameter diversity) and Cooperative forecasting (pre-processing and post-processing). The recent research papers that have been selected which have been published in the last four years (2018–2022) were reviewed. The above-mentioned papers focused on reviewing solar forecasting methods. In this paper, the focus was ensemble forecasting methods and their classifications in recent years.

## Ensemble Forecasting

**Fig. 3:**
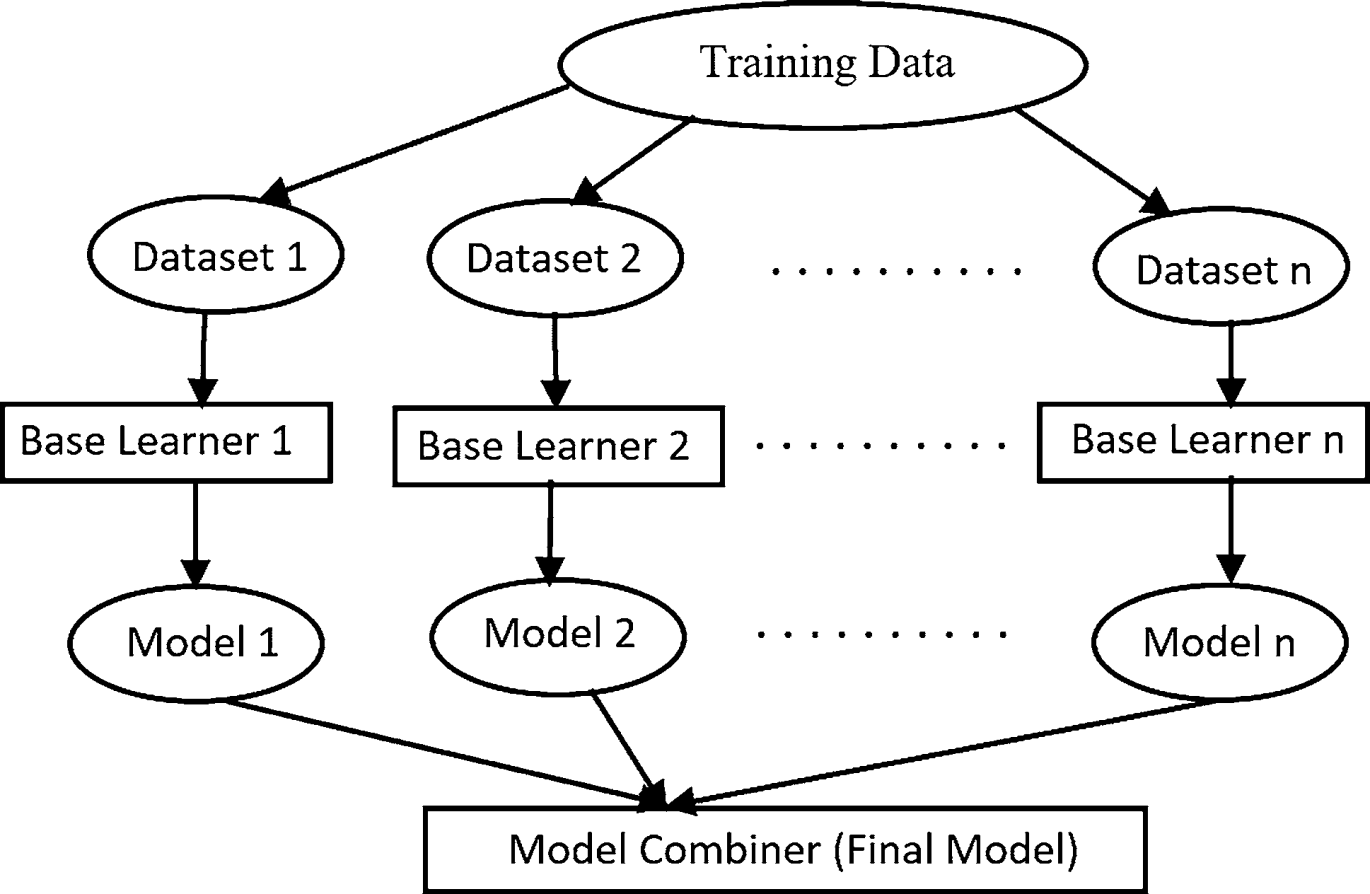
General construction flow of an ensemble model

The main concept of the ensemble technique is training ensemble members (base learners) and combining their prediction into a single output to obtain a better performance of a model [10]. Figure 3 shows the typical ensemble model construction. As can be seen in the Fig. 3, the training data is divided into many data sets initially and then several base learners are generated which can be run in a sequential or parallel format. Finally, the combination of the models is run on the base learners [43, 44].

On other words, Ensemble (hybrid, or combined technique) [45] is a machine learning-based method in which multiple predictors are used to reach an aggregated decision in a better format of base predictors. The main advantage of an ensemble model is that it incorporates the suitability of its constituent techniques, which creates a stronger learning pattern. So, it can enhance the accuracy and strengths of individual methods by solving their weakness [46]. The ensemble methods can be classified into two main groups: competitive and cooperative [39]. In a competitive ensemble forecasting, the different base predictors train individually with the same or different data sets by using different parameters, and the prediction is obtained from the averaging the decisions of all base predictors [47]. Besides, in a cooperative method, the prediction task will be divided into several sub-tasks to select the appropriate predictors based on the characterization of the sub-tasks. Then, the sum of all the base predictors’ output will be the final decision of the cooperative method [48]. Table 2 shows the classification of the ensemble methods and their perspectives in this paper.

**Table 2:** The classification of ensemble forecasting models [49]

Categories	Perspectives	Methods
Competitive ensemble methods	–Data diversity –Parameter diversity	– Bagging, Boosting – Ensemble Kalman filter
Cooperative ensemble methods	–Pre-processing –Post-processing	– Wavelet decomposition (WD), empirical mode decomposition (EMD) – ARMA-GARCH, ARMA-ANN, ARIMA-SVM

### Competitive Forecasting Methods

As mentioned earlier, a competitive forecast model can construct individual forecast models for the formation of an ensemble forecast model by using multiple predictors using different initial conditions or parameters. Then, the results will be obtained from the average of the selected models [50]. The competitive ensemble methods are categorized into three different perspectives which are data diversity, parameter diversity and structural diversity [51]. Diversity is the most important feature of competitive ensemble forecasting. For instance, the outputs of base predictors would be similar if the sub-tasks are similar while the performance improvement of ensemble predictor will be marginal [50]. Some examples of competitive ensemble forecasting models are discussed in detail in the following sections.

#### Data Diversity

Data diversity is another category of cooperative ensemble forecasting method in which the forecasting system is fed by more than one input dataset [37]. As shown in the following equations, two variations have been used in Eq. (9) and (10). Equation (9) applies N predictors (${f}_{1}\left(0\right)\dots {f}_{N}(0))$ for N input datasets ( ${x}_{1}\dots {x}_{N}$) for forecasting and the weighted average of all of them will be the final prediction value, but Eq. (10) uses only a single predictor for forecasting.

8 $$\hat{y}(t + h) = \frac{1}{N}\sum\limits_{i = 1}^{N} {w_{i} f_{i} (x_{i} (t))}$$

9 $$w_{i} \ge 0$$

10 $$\sum\limits_{i = 1}^{N} {w_{i} } = 1$$

11 $$\hat{y}(t + h) = f(x_{1} (t),x_{2} (t),...,f_{N} (t))$$

where $\widehat{y}$ is the predicted value and $h$ is the forecast horizon. The most famous and common approaches are bagging and boosting for data diversity [48].

##### Bagging

**Fig. 4:**
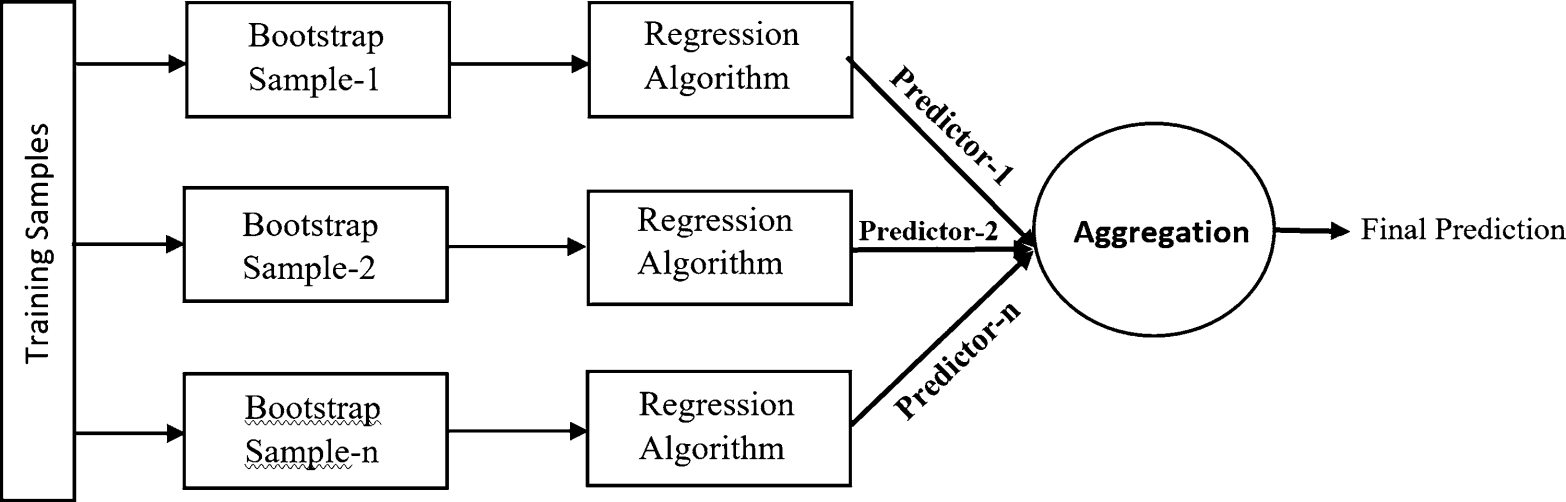
Block diagram depicting the basic principle of Bagging ensemble learning

For the first time, bagging or bootstrap aggregation is proposed by Breiman [52] which helps decrease the forecasting model variance and avoid overfitting [51]. The main goal of Bagging is developing several estimators, and results are obtained by aggregation of individual estimators' results with some biasing. Initially, from the original datasets, several subsets of training data are created. Then, the samples are selected randomly by replacement of data samples which are called Bootstrap samples. In the last step, the final prediction is obtained by aggregation of all the bootstrap predictions [53]. This process is shown in the Fig. 4. One of the advantages of bagging is the error reduction of ensemble generation in the baseline predictors. It also can correlate its estimation with real datasets using estimates of test sets or cross-validation [54].

**Fig. 5:**
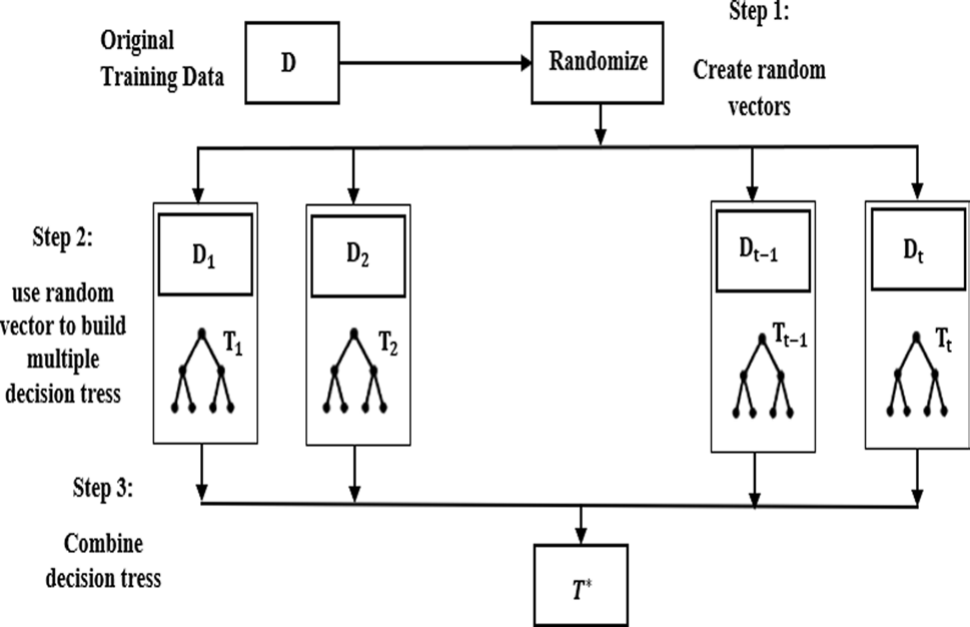
The procedure of random forest method [59]

The most common algorithm for bagging is random forest (RF) which can be considered as an extension to the bagging concept, and it can be used for classification and regression. It is made up and trained a large number of decision trees (DT), called predictors and each one produces their own predictions that can create higher accuracy in prediction [55, 56]. In the RF algorithm, several decision trees are constructed through training samples (a subset of training samples are injected into each tree randomly). The output of RF is obtained by voting of the decision trees. In the case of classification, the majority of voting is used to decide on the predicted result, but in the case of regression, the mean value of the predictions of all the estimators (predictors) are calculated [57]. Figure 5 demonstrates the procedure of random forest method graphically. This algorithm does not need complex calculations like Support Vector Machine (SVM) and ANN because the main variables that should be adjusted is the number of trees. Also, compared to ANN and SVM, training process is faster in the RF. In this algorithm, if sufficient trees are used, it is robust to noise and outliers [58].

The application of this RF can be extended to PV production forecasting. Tato et al. used real radiation measurements by combining simple radiation predictions to forecast the solar energy output for short-term temporal horizons [60]. A daily PV power generation forecasting model was proposed for North China in winter. The proposed forecasting model was based on the RF algorithm using weather measures [61]. The accuracy, extra trees (ET), computational cost, and stability of RF were investigated for predicting hourly PV generation output. In addition, their performance was compared with support vector regression (SVR).The performance evaluation of the model was performed by RMSE,MAE and ${R}^{2}$ and it was concluded that RF performed better on both testing datasets and training in comparison with other models [62].

##### Boosting

To solve the classification and regression problems, boosting can be also used as a powerful learning strategy. It can be done by the combination of weak learners’ output into a ‘committee’. In addition, boosting improves the suitability of model to data through bias reduction [63]. In boosting process, several subsets can be created from the original dataset. Then it sequentially trains the predictors with the datasets, and at each iteration, smaller weights to the data points with smaller error and higher weights to the data points with larger error can be assigned by boosting. Finally, the results from the weighted average will be obtained [64]. Different algorithms can be used in bagging methods such as AdaBoost [65], extreme gradient boosting (XGBM) [64], gradient boosting machine (GBM) [66, 67], and light gradient boosting machine (LGBM) [68]. The recent advances in boosting algorithms and their applications in energy research such as solar and wind have been reviewed in [69]. They showed that how boosting algorithms are effective tools in the performance of prediction models.

Recently published studies are using bagging and boosting methods for forecasting solar irradiation in different regions. For instance, to improve forecasting accuracy in solar energy output, the bagging model was used in [64]. The proposed model as an ensemble method, used a based learner such as random forest, LGBMs, and XGB by addition of the past output data as new features. They found the bagging model was successful with higher model accuracy using past data features in comparison with a single model learner with default features [70].

In another study [44], the solar irradiance in five cities of Turkey, was estimated using bagging and boosting ensembles of ANN, SVR, and DT. Initially, base models (ANN, SVR, and DT) were created and examined by using 5 years of meteorological data. Then, both bagging and boosting ensembles methods of the base models were constructed and tested with the same data. The results were compared based on two evaluation metrics namely RMSE and ${\mathrm{R}}^{2}$. Based on their findings, the proposed model based on bagging and boosting methods improved ANN, DT, and SVR in the range of 4.6 and 14.6% in terms of RMSE. Several ensemble models to predict short-term solar irradiation were investigated in [71]. The models were RF, Boosted Trees, Generalized Random Forest, and Bagged Trees. The performance of these methods was validated via ${\mathrm{R}}^{2}$, and MAPE and compared with SVR and Gaussian process regression. Their result showed a consistent and reliable prediction of ensemble methods with high prediction performance in comparison with the individual regressors. In a recent study in 2022, a natural gradient boosting (NGBoost) algorithm was used for the short-term solar prediction of PV power systems based on physical properties and human intuition. They found a 6% increase in RMSE with apply the most important features of the ensemble method [72]. The several tree-based methods of DT, GBM, XGBM, bagging, RF, and Cubist [73, 74] were used for forecasting solar irradiation on Jeju Island of South Korea. Also, the prediction performance was based on the comparison of MAE, RMSE, and nRMSE [75].

#### Parameter Diversity

In contrast to data diversity, parameter diversity is applied different parameter settings with the same dataset $(x)$. The forecasts can be generated by using the following equation [71]:

12 $$\hat{y}(t + h) = \frac{1}{N}\sum\limits_{i = 1}^{N} {f_{i} (x_{i} ,\theta_{i} )}$$

where $h$ is the forecast horizon and $\widehat{y}$ is the predicted value.${\theta }_{i}$ is the parameter for model${f}_{i} , i=1,\dots , N$. It should be noted that the combination in Eq. (11) uses equal weighting. In solar forecasting studies, once the initial conditions need to be perturbed, parameter diversity is a useful method as it considers numerical weather prediction models (NWP) [76, 77]. Kalman filter is the best method for NWP which estimates the “true” state of a dynamical system from noisy measurement data [76]. The Kalman filter processes in two stages including prediction and updating. In the first stage, there is a current state vector contains one or more state variables, and the state vector with a weighted average will be updated in the second stage. In addition, without additional past information the Kalman filter can run in real-time due to its recursive nature [78]. Recently, studies have been focused on solar forecasting using Kalman filtering.

Yang studied day-ahead NWP forecasts using Kalman filtering to forecast solar irradiation. The author used multiple Kalman filters to maintain the original day-ahead horizon [79]. Besides, an ensemble version of Kalman filter is called Ensemble Kalman Filter (EnKF) which can correct the forecasted value in real-time. It can be done by propagating the uncertainties in time [80]. In another study, the Ensemble Kalman filter (EnKF) and the state-space models (SSMs) in a short-term PV forecasting experiment which was effective to forecast solar irradiance [81].

Jiranantacharoen and Benjapolakul [82], used Kalman filter and Auto-Regressive Integrated Moving Average (ARIMA) for forecasting photovoltaic (PV) power generation. It should be noted that real-time measurement data is needed for Kalman filter to adjust forecast value. Then, they proposed a predictor model to apply in the forecasting process once the real-time measurement data is unavailable. In addition, to estimate the transition matrix for running Kalman filter, ARIMA model was applied and the model performance by RMSE and SS.

### Cooperative Forecasting Method

The cooperative ensemble forecasting is another type of forecasting method which divides a prediction task into several sub-tasks. These tasks can be solved individually. In addition, the output of one sub-task can be considered as input into another sub-task. The overall process of cooperative ensemble forecasting is distributed into two assignments. The first one is to realize the prediction task and the second are some assistant procedures such as parameter optimization, preprocessing, error correction, feature selection, and postprocessing.

Preprocessing includes reconstruction, decomposition, and transformation which should be performed before forecasting [49, 83].

#### Pre-Processing

Raw renewable energy data always has a variety of irregularities, such as fluctuations. These irregularities have nonlinear and non-static properties which deteriorate the performance of the forecasting. Therefore, many pre-processing techniques have been proposed to break down the renewable energy original signal into several components. These techniques have better behavior in terms of outliers and data variance. With the help of these data processors, the negative impact of irregularities on the accuracy of forecasting can be properly reduced [84]. In pre-processing forecasting method, the data is decomposed into finite numbers of subseries. It is noted that the performance of each subseries is better than the original data. Then, to forecast each subseries, a regressor and feature extractor is developed independently. In the next step, all the subseries is combined, and the forecasting results will be generated. In pre- processing forecasting, Empirical mode decomposition (EMD) and wavelet decomposition (WD) are two of the most widely used methods.

Time series of temperature data and solar irradiance include seasonal long-term behaviors, and daily information. Then, for training, it is suggested that to improve the forecasting model performance, the frequency contents of those signals can be used instead of the signal values. To do this, the forecasting models can be based on WD of the input data [85]. The WD deal with the solar irradiance fluctuations and used to the input data of a forecasting model and, which resulted in the accuracy improvement. Generally, an efficient solution have been made using the wavelet techniques represents resulted in the noise reduction in input datasets before to implement a prediction model [86]. Some recent studies about WD have summarized (based on the time) as follows.

A method combining the ANN and WD for the forecasting of the power output of PV power plants was presented by [87]. The solar irradiance, and meteorological variables such as wind speed humidity, and temperature were chosen as inputs of the ANN model as the ANN cab address their nonlinear relationships. Then, WD is used to decompose output power of the PV plant resulted in the separation of the useful information from disturbances. To build models of the decomposed PV output power, ANNs were used. Next, various sky conditions include rainy, sunny, cloudy days and overcast were proposed for validation of model. Finally, the ANN applied to compare the presented method with the traditional forecasting method. The results show that the method needs lesser calculation time with a better forecasting accuracy (MAE of 10.34%, MAPE of 25.37%, and RMSE of 19.66% in rainy days).

Ref [88] was proposed a model called wavelet-coupled support vector machine (W-SVM) in global incident solar radiation forecasting based on the minimum and maximum temperature, sunshine hours, evaporation, precipitation and wind speed as the predictor variables. The merit of the W-SVM was benchmarked with the classical SVM model to achieve reliable results. Then, in sixteen months from 01-March-2014 to 30-June-2015 the data were divided into the test (35%) and train (65%) were set for daily forecasting in the three metropolitan stations (Townsville Aero, Cairns Aero and Brisbane City). The forecast was assessed by prediction errors (MAE, RMSE, MAPE and RMSE). Based on the obtained results, the W-SVM model outperformed better than the classical SVM model for daily forecasts using optimum input combinations.

By referring to Ref [89], the merits of wavelet-ANN models for solar radiation was evaluated. Four different architectures of ANN, namely: adaptive neuro-fuzzy inference system (ANFIS), generalized regression neural networks (GRNN), nonlinear autoregressive recurrent exogenous neural network (NARX) and multilayer perceptron (MLP), were used. The for the decomposition of the complex meteorological signals into relatively simple parts a wavelet analysis was used by using wavelet sub-series, and WD transformation algorithm. The ANN models were used to model the wavelet sub-series and reconstructed to estimate the original signal. Then, to model the global horizontal irradiation over Abu Dhabi city, four meteorological parameters were used including temperature, wind speed, relative humidity, and sunshine duration. The proposed approach was compared to ANN models and validated using different metrics such as ${\mathrm{R}}^{2}$, t-statistics RMSE, MAPE, and MBE. The results confirmed the proposed model improved the performance of the ANN with a maximum 6.84% in ${\mathrm{R}}^{2}$ for MLP meanwhile GRNN had a minimum of 2.78% RMSE.

In [90], the information from raw data with better time–frequency resolutions was extracted and the WD was applied with a bias compensation Random Forest (BCRF) to minimize the prediction error. First, eight decomposition layers in a stationary WD on all raw input features was conducted. Then, there was the lower frequency part of the original signal due to the higher level of decomposition. Next, to train a random forest regression model, the wavelet components and time index as input features were used. In fact, an additional model created by BCRF for prediction of bias to minimize the overall prediction error. Finally, Wavelet – BCRF technique was evaluated by some error metrics like RMSE, MAE and MAPE.

In another study [91], a multi-level WD based on day-ahead solar irradiance forecasting method was proposed. Initially, based on the weather conditions, the daily solar irradiance series were classified into different patterns. Next, the solar irradiance of the next day 24 h was forecasted for each weather pattern using decomposed data series at different WD levels. Then, to fuse the predictions into the final forecasting output, a data-driven fusion model corresponding to the weather pattern was applied. Simulations showed that the forecasting accuracy using different WD level data depends on the weather conditions (sunny, cloudy, and rainy days). In sunny days, solar irradiance reached its maximum level, and the day irradiance curves were relatively smooth but in cloudy and rainy days the value of solar irradiance was limited to lower level and there were more fluctuations on the irradiance curve. To evaluate the forecasting accuracy, two error indexes of RMSE and MAE were used. Generally, the forecasting accuracies all showed the trend of first increasing and then decreasing with WD.

It is usually difficult to design and implement forecasting for non-stationary and non-linear signals. In these cases, The Fourier decomposition can be used, but it does not give information about the time scale characteristics of the data. For this purpose, and in order to obtain the time-scale (time–frequency) information of the signal, we need a method that can extract the intrinsic modes embedded in the signal [92]. The EMD method is proposed for this purpose. In fact, the EMD is a signal analysis method that can indicate at what moment, at what frequency, and with what intensity it is present in a signal [93]. By EMD, there is the decomposition of complex signal into a linear combination of a limited number of intrinsic mode functions (IMFs) with different frequencies. Hence, each of the decomposed IMF components contains local characteristic signals of different time scales from the original signal [94]. Some recent studies about EMD have been summarized as follows.

In [95], a hybrid EMD and back-propagation neural network (BPNN) model was developed for photovoltaic power forecasting. Then, each IMF and each residue were used to train and test the BPNN individually after decomposition of the time series data by EMD. The proposed EMD-BPNN model was evaluated with PV power output time series data. These data were collected from grid-connected photovoltaic power plants situated in Ghaziabad India at the 100-kW roof-top. Finally, for the performance evaluation of the developed model, the data set was divided into the four evaluation parameters (the symmetric mean absolute percentage error (sMAPE), MSE, MAPE and RMSE) and weekly data groups (W1-W4) with different forecast horizon of 1, 12 and 24 h ahead. Results indicate that the data decomposition greatly reduces the complexity and evaluation time of the back-propagation neural network.

In another study, a hybrid model with combination of ensemble empirical mode decomposition (EEMD) and variable weights was proposed to overcome the demerits of the EMD-ANN model. Therefore, for decomposition of the original PV power generation data, EEMD was used to obtain the residual component (RC) and multiple IMF components (IMF_1, IMF_2). Then, residual components and IMF components were divided into low-frequency, intermediate-frequency, and high-frequency sequences. Then for the prediction of these three sequences, variable-weight combination forecasting (VWCF) method was used. The final prediction results were calculated by summation of the three forecasting results. The total number of input variables were 13 but the first six influential variables were top net solar rad, surface thermal rad down, temperature, surface pressure, time, and relative humidity. RF algorithm was used for the determination of each variable impact. Then, MAE and MSE were used for the evaluation of the forecasting results. Based on the results, the prediction accuracy of each model (in terms of MAE) was reduced [96].

In [97], the pre-processing technique (EMD) was presented to decompose the data. The solar power output using the hybrid design of the SVR model was used the improved feature selection algorithm that resulted in the selection of the best input for the next processing. To improve the accuracy of the model, the proposed model design was set based on the SVR with PSO optimization. The results showed that the proposed algorithm performed better with an average of 14.55 (%) of MAPE and 0.95 (%) of nRMSE.

In [98], several multiscale decompositions in methods of time series analysis for one-hour global solar radiation. Initially, they calculated the time series of the Clear Sky Index. Then, EMD and EEMD methods are used to decompose obtained time series data. Next, the data was forecasted using a linear model and nonlinear models using the time scale fast fluctuation components. Finally, the results were improved with a combined hybrid model using globally multiscale decomposition.

In [98], several multiscale decompositions in methods of time series analysis for one-hour global solar radiation. Initially, they calculated the time series of the Clear Sky Index. Then, EMD and EEMD methods are used to decompose obtained time series data. Next, the data was forecasted using a linear model and nonlinear models using the time scale fast fluctuation components. Finally, the results were improved with a combined hybrid model using globally multiscale decomposition. Table 3, which summarizes the above, is in the appendix.

**Table 3:** Summary of recent studies for solar forecasting using perspectives

Ref	Time ahead	Input variables	Output variable	Perspectives	Forecasting method	Error	Comparison
[53]	Daily	−Wind Speed −Wind Direction −Air pressure	Wind power	Data diversity	Bagging	RMSE = 10.6%	Bagging > ANN > k-NN
[61]	Daily	−Temperature −Air Pressure −Wind	PV power	Data diversity	Random Forest	MAPE = 8.5%	RF > GBDT
[63]	Daily	−Temperature −Relative humidity −Cloud cover −Precipitation	PV power	Data diversity	Boosting	RMSE = 9.48%	Boosting > AR
[65]	Hour	−Wind Speed	Wind power	Data diversity	Ada Boosting	MAE = 1.0581 MAPE = 7.63%	AB > ANN
[76]	Daily	−PV power	PV power	Parameter Diversity	Time series	MSE = 16.24	TS > SARIMA
[77]	Hour	−Irradiation	PV power	Parameter Diversity	SVR	MAE = 37.04	SVR > NAM > SP
[81]	Hour	−Irradiation −Temperature	Purchased PV power	Parameter Diversity	Polynomial Regression	MAPE = 10.51%	–
[87]	Daily	− Humidity − Temperature − Wind Speed	PV power	Pre- processing	WD-ANN	RMSE = 19.663% MAE = 10.349%	WD-ANN > ANN
[88]	Daily	− Sunshine hours − Temperature − Wind speed—evaporation—precipitation	Global solar irradiation	Pre- processing	W-SVM	RMSE = 2.317 MJ ${m}^{-2}$ MAE = 1.819 MJ ${m}^{-2}$	W-SVM > SVM
[90]	6 h	− Solar irradiance − Temperature − PV output	PV power	Pre- processing	WD-BCRF	RMSE = 32.12% MAE = 20.64%	WD-BCRF > WD-SVM > RF
[96]	1 h	− surface thermal rad down − Top net solar rad − surface pressure − Humidity −Temperature	PV power	Pre- processing	EEMD-VWCF	RMSE = 0.0107 MAE = 0.0622	EEMD-VWCF > BPNN > RF
[95]	1,12 and 24 h	−	PV power	Pre- processing	EMD-BPNN	RMSE = 0.019 MAPE = 5.47%	EMD-BPNN > BPNN
[38]	10 min	− Irradiation	PV power	Post- processing	ANN	RMSE = 6%	ANN > ARIMA

#### Post-Processing

In general, PV data have time-series measurements [99]. In post-processing as a cooperative ensemble forecasting method, the forecasting will be performed using the time series consecutively by two or more predictors. Moreover, a time series data may have more than one characteristic which is suitable for one specific method. For instance, ANN is usually applied for modeling non-linear time series while ARIMA model is suitable for modeling linear time series [38]. The main advantage of post-processing methods is the improvement in the bias of the global solar irradiance forecasts without the need for a long-term historical data database [100]. Based on post-processing method, there are several cooperative ensemble forecasting models such as ARIMA-ANN, ARIMA-SVM, ARMA_ANN, ARIMA-GARCH, SARIMA-ANN, and ARMA-GARCH.

Based on the information, post-processing methods are often used to optimize the output of NWP models. It should be noted that detailed local weather characteristics generally cannot be resolved by NWP predictions hence the spatial resolution has been grown recently. By using the post-processing method, the forecasting will be improved slightly by consideration of the uncertainty of some forecasts even though most of the forecasting methods contain statistical components. Therefore, the final forecasting of data using this method will be a single value that can be valid for future studies [101].

In a study by David et al., two models of ARMA and GARCH were combined to provide probabilistic forecasts of solar irradiance. Meanwhile, to provide a framework that can be applied in an operational context easily, a recursive estimation of the parameters of the models has been set up. As they found, higher forecast accuracy has been found by the proposed method (ARMA and GARCH) in comparison to other machine learning-based techniques. In addition, this model gave additional information about the uncertainty of the forecasts which was easier to set up [102].

A solar radiation forecasting time series model was proposed for multi-hour forecasting (915 h ahead) and a small-scale solar radiation database (30- and 1-s scales) for 1 day (47,000 s ahead). In the first step, ARMA was used to predict future values of the global solar radiation time series. Then, a nonlinear autoregressive (NAR) neural network was applied for prediction purposes due to the nonlinearity in solar radiation time series. The results showed that the ARMA- NAR combination had better accuracy. The NRMSE of the hybrid model was equal to 0.2034 compared to 0.3241 for ARMA model and NRMSE equal to 0.2634 for NAR model [103]. An innovative hybrid model was proposed in three different cities in Morocco for forecasting the daily global solar radiation. There were three steps for estimation including 1) evaluating the linear aspect of the problem by ARIMA model, and 2) building an ANN model to model the residuals of the ARIMA. It was estimated that the error conditions for the ARIMA model can be forecasted by output from ANN model. The findings showed that the hybrid model (ARIMA–ANN) was more accurate in terms of MAPE, R2, RMSE, MBE, NRMSE, and TS [104]. Table 2 shows the findings of recent studies that use ensemble methods for solar forecasting.

## Conclusion

In general, the reliability of solar power systems is affected by the dynamic nature of solar irradiance. Changes in sunlight intensity led to voltage and power fluctuations in solar power plants and disruption of power systems. A good way to deal with such problems is to predict solar irradiance. Accurate forecasting is challenging and involves a variety of methods statistical, physical and ensemble forecasting methods. This paper has reviewed recently published studies (2015–2021) on solar irradiance forecasting using ensemble models including competitive and cooperative forecasting methods. The former has been divided into data diversity and parameter diversity and the latter has been categorized according to pre-processing and post-processing. In this paper, recent articles have been discussed according to each category. It can be concluded that ensemble models perform better than standalone ones. However, a hybrid models have a more complex structure, but they provide better accuracy. Based on the previous studies, Artificial Neural Network (ANN) and Space Vector Machine (SVM) are widely used with ensemble models (WD-ANN, EMD-BPNN and W-SVM) due to their ability in solving complex and non-linear forecasting models. Also, the performance of the ensemble models has been evaluated by some error metrics such as RMSE and MAE. It has been indicated that EMD is more powerful, and it has more accuracy than WD in solar forecasting methods.

Moreover, in this article, the different model inputs, and their effects on the prediction of solar radiation have been discussed. Common inputs are solar irradiance, atmospheric and module temperature, wind speed and direction, and humidity. Among these, solar irradiance is most positively correlated with PV output. Solar irradiance is positively correlated with temperature and negatively correlated with wind speed. Other weather variables have low correlation values.

## Appendix

See Table 3.
